# Technology clinical trials: Turning innovation into patient benefit

**DOI:** 10.1177/20552076211012131

**Published:** 2021-04-30

**Authors:** Jennifer K Royle, Andrew Hughes, Laura Stephenson, Dónal Landers

**Affiliations:** 1 digital Experimental Cancer Medicine Team, Cancer Research UK Manchester Institute, University of Manchester, Manchester, UK; 2 Division of Cancer Sciences, University of Manchester, Manchester, UK

**Keywords:** Patient, carer, technology, digital, device, clinical trial, methodology

## Abstract

Health care needs to continuously evolve and innovate to maintain the health of populations. Technology has the potential to enable better patient engagement and ownership, as well as optimise therapeutic interventions and data-science approaches to facilitate improved health care decisions. Yet, to date, technological innovation has not resulted in the rate of change that could have been predicted from other sectors. This article discusses multiple reasons for this and proposes a newly tested and deployed solution: the technology clinical trial. The technology clinical trial methodology has been developed through working directly with patients, clinical and medical devicetrial experts. This approach enables researchers to use the complex environment of health care as an opportunity to transform the pace of innovation and create new care pathways. Instead of testing a single innovation, researchers can ‘step back’ and systematically review all areas of the patient's journey for potential optimization. Then integrate novel data science, technological advances, process updates, behavioural science, and patient engagement to co-create a streamlined multidisciplinary solution. As a result, this research has the potential for larger advances due to the emergent benefits that can arise when the individual elements work together as a whole. These potential benefits are then robustly tested, characterised and measured in the trial environment to ensure that future application of the innovative pathway is supported by the robust empirical data health care requires.

## Background

Health has a direct impact on both the economy and each person’s individual enjoyment of life. Countries around the world are facing the monumental problem of maintaining the health of aging populations through either prevention or treatment of disease. Health promotion and prevention campaigns are prevalent, varied, and have had differing success over the years.^1–4^ Non-communicable diseases (such as diabetes, cardiovascular or chronic respiratory disease, or cancer), however, remain responsible for roughly 60 percent of all deaths and nearly half of the loss of actual and effective life-years due to disability and death.^5^ New therapeutic options must therefore be developed to improve health.

Clinical Trials of Investigational Medicinal Products (CTIMPs) test new drugs and ensure that they provide acceptable gains in benefits, and reductions in risks, when compared with existing options. Medicines themselves, however, are only one aspect of disease treatment. Health care is the point where clinical-science meets people – and it is usually people who decide how, and whether, they will accept the proposed therapeutic intervention. The widespread acceptance of the internet, mobile technologies, home test-kits and sensors provide the opportunity to revolutionise the way that patient care is delivered. Traditional clinical trial approaches can be supplemented and adapted using innovative data-science and technology to improve the timeliness, accuracy, and personalization of decisions, as well as the way people can engage-with and support their own health.

This paper identifies four key elements that underpin all current care pathways and must be addressed by research for future innovation to robustly realise its potential:

- Human expertise: People involved in health care are highly trained specialists in their fields.
- Science: Including treatments, and data-science to facilitate recovery from illness and/or improved quality of life.
- Technology: Found throughout health care settings with examples from data collection, storage and protection, through to drug delivery, decision-support, or robotics.
- Human behaviour: Health care is delivered and received by people. Outcomes are ultimately shaped by the behaviours and choices these people make.

Optimization of a care pathway requires integrated improvement in all four areas and does not solely rest upon the new drug or a single device. Adaptive clinical trials and adaptive interventions have already employed a broader-scope of innovation through integrating improvements in both data-science and trial methodology into decisions.^6,7^ However, even though these initial steps have been taken, the mechanics of clinical-trial delivery, as well as the remits of those involved, remain fundamentally unchanged:

- Regulators ensure that legal and ethical standards are upheld.
- The trial-sponsors design the trial to research new drugs or a new medical device whilst ensuring appropriate safety-monitoring and mitigation requirements are in place.
- Hospital staff deliver patient care in time-pressured environments that conspire against timely collection and reporting of trial-data for sponsors.
- Patients themselves are asked to comply with requirements outlined by their health care team and share information when asked (verbally or through testing).

This delivery model reflects a traditional top-down, paternalistic approach to delivering health care in which clinical teams are cast as expert providers whilst patients are cast as grateful recipients, with most information-exchange occurring when the patient visits their clinician.^8^ Nevertheless, hospital visits – though often essential – can only provide a snapshot of someone’s remembered experience, and are therefore subject to recall bias.

Technology has the potential to transform this established model through evolution of patients' roles, increasing the breadth of data-capture so that patients' experience can be fully understood, whilst enabling continuous feedback between patient's and clinical teams, and thereby improving interpretation of data to deliver increased patient benefit. To date, technology and data-science innovations have not resulted in the rate of change that could have been predicted when compared to other sectors.^9,10^

Many reasons have been proposed to explain this slow acceptance into the health care systems, however, all fundamentally rest on the medical principal of *primum non nocere* (first, do no harm). Device regulations, and therefore trials, focus upon gathering evidence to demonstrate that new medical devices are accurate, reliable, safe, and do the job they are designed for. However, nothing in health care is used in isolation and technological-advances have the potential to change elements far beyond use of the device itself – reaching into roles, medic-nurse-patient-carer relationships, behaviours, and health care culture.

**Figure 1.**
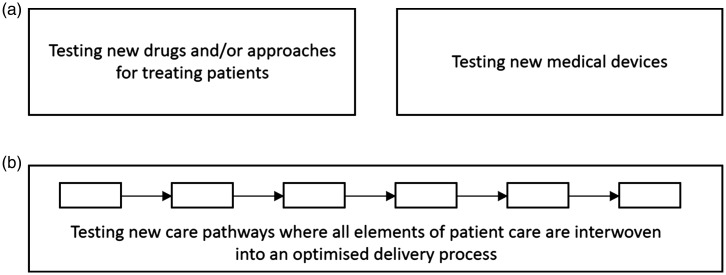
Established and new methodology fields in the clinical trial landscape. (a) Established fields, CTIMPs or medical device studies, each normally focused on assessment of a single intervention (drug or device), or combined intervention (drug and device). (b) New field of clinical research, assessing the patient benefits from innovation in an integrated care pathway. Fills the gap that currently exists between CTIMP and medical device studies and supports subsequent uptake and adoption of the approach - the so called “Technology clinical trial”. CTIMP: Clinical trials of investigational medicinal product.

There is understandable caution when people are upholding the principle of ‘do no harm' and yet the broader care-pathway change-requirements of these innovations have not been tested. There exists the requirement for a field of clinical trial research that is different from traditional focused CTIMP or medical device trials (Figure 1). In this research, the potential of technology and data science is used to strip a care system (pathway) back to its essential components, integrate novel innovations, and then redesign all four elements of the approach to optimise patient benefit. Such methodology has the potential to change the fabric of care itself. These new, optimised, care pathways are then objectively tested in a ‘Technology Clinical Trial' so that future care-deliverers have the data to know whether the approach works, how to operate each element of it, and understand the quantified benefits and risks that come with the broader care pathway modifications both hospitals and clinical trials depend upon.

A central question for technology clinical trials is ‘who is best placed to deliver them?'. Drug-sponsors are experts at creating and delivering CTIMPs and protocols, however they do not traditionally modify underlying hospital processes and are, consequently, not usually best placed to develop and deliver this integrated trial methodology. Similarly, it is rarely appropriate for medical device manufacturers – who are frequent device-validation trial sponsors – to confuse and encumber their device-specific trial results with broader pathway modifications. Medical teams themselves have the potential for delivering technology clinical trials, with the power to transform care pathways, but rarely have the spare resource with service-provision in busy hospitals. However, academic research groups, especially those functioning within both the hospital and academic networks, are ideally placed to deliver cross-disciplinary technology clinical trials and further support evidence-based medicine. The problem halting their progress to date has been one of methodology: how to create new pathways? And how to deliver trials in such a complex regulatory and legal landscape?

The digital Experimental Cancer Medicine Team (digital ECMT) in Cancer Research UK, Manchester Institute, University of Manchester have developed methodology to address these issues in practice and are successfully delivering technology clinical trials. This article aims to share this novel methodology with other research groups to support broader adoption of objective care-pathway assessment through the application of technology clinical trials.

## The technology clinical trial approach

**Figure 2.**
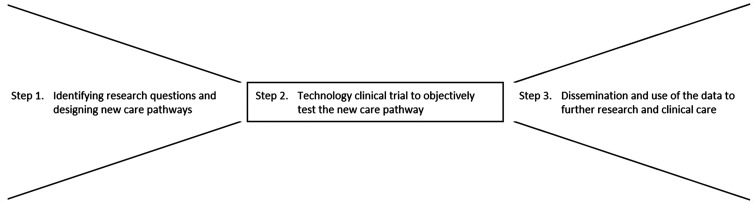
Overview of methodological steps for developing and testing new clinical care pathways. Step 1. Co-creation of care pathways and addressing information governance, data protection, regulatory and ethical questions by design. Step 2. Delivery of the technology clinical trial. Step 3. Supporting future research and uptake in practice.

The methodological approach to developing and testing integrated care pathways involves three steps: Step 1, identifying research questions and designing new care pathways; Step 2, technology clinical trial to objectively test a new care pathway; Step 3, dissemination and use of the data to further research and clinical care. Together these form a learning cycle that develops ideas into practice and evolves with emerging science (Figure 2).

### Step 1. Identifying research questions and designing new care pathways

**Figure 3.**
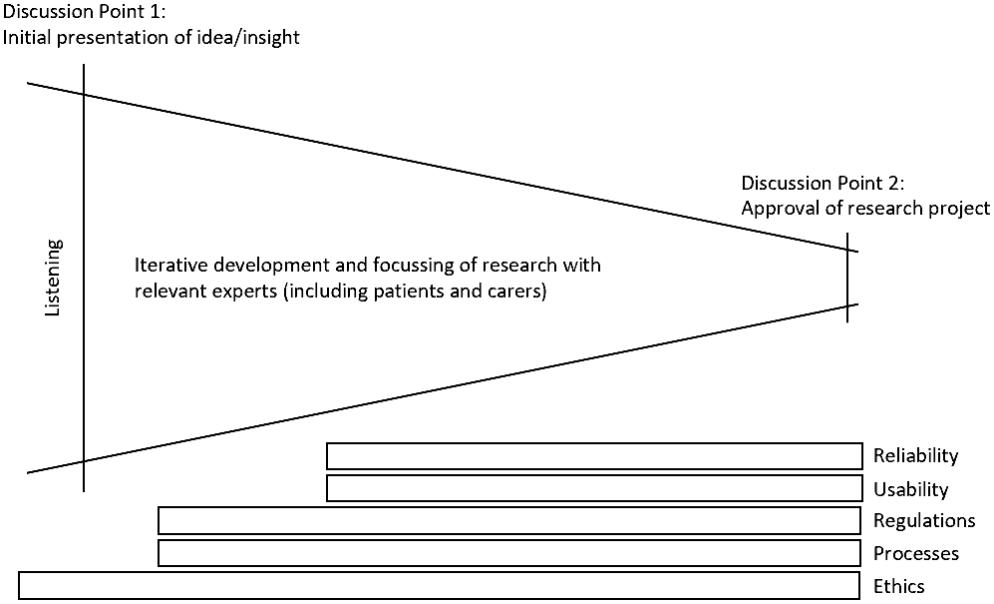
Translation of initial ideas into research projects. Process for ensuring most relevant research is supported and that solutions are co-created to meet the requirements of users, ethics, regulations, information governance and privacy.

The secret to identifying new projects is to talk with the people involved, to watch, listen, and stay curious. For this purpose, the digital ECMT has a design-lab within Manchester’s National Institute for Health Research (NIHR) Clinical Trials unit at The Christie National Health Service (NHS) Foundation Trust. Not only are technological solutions supported from the design-lab, but the room provides a space to directly interact with both staff and patients/visitors to the hospital trials facility. Interactions are documented according to the verbal consent of those taking part in the discussion, and anonymised information captured. This provides an evolving insight into the needs of those involved in clinical trials at site. The needs of trial sponsors and device manufacturers are generally gathered either via network discussions, collaboration, or through conference attendance. The research group therefore maintains a constantly evolving awareness of the largest recognised opportunities for improving patient benefit. These opportunities are shaped into research questions that are then explored and honed into new care pathways (Figure 3).

In this methodology, research questions are first debated during ‘Discussion Point 1’ (DP1) meetings. The role of DP1 meetings is to use the broad expertise available to the research group when devising ways of potentially addressing the problem. Anyone with relevant insights to the new process is invited and all are addressed as equals and experts in their area. Uniqueness of the opportunity is assessed, known information characterised, required knowledge and key ethical discussion points are documented,^11,12^ and potential approaches listed as starting points for solution (care pathway) iteration. This drives the creation of a project plan for feasibility scoping that can be undertaken by the team.

**Figure 4.**
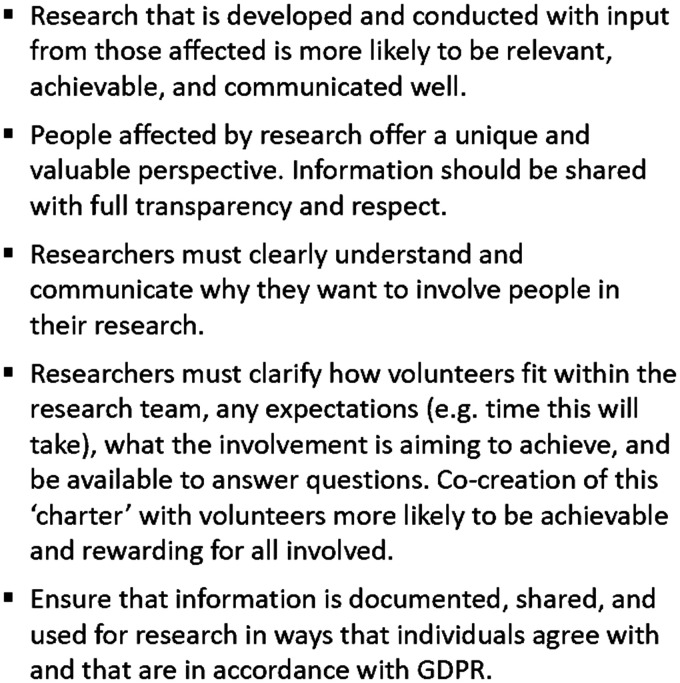
Key involvement principles. Key patient and public involvement principles. Further information can be accessed via the National Institute for Health Research INVOLVE guidance,^13^ the Health Research Authorities' public involvement webpage, or using the Cancer Research UK toolkit.^14–16^ GDPR: General Data Protection Regulation.

Input is required regularly during these early exploratory stages of the project, with the design-lab in hospital proving essential for staff, patient and carer involvement. Various forms of engagement are utilised for solution-iteration including focus groups, in-clinic discussions, and hands-on operation of solutions. At all times, the principles of the NIHR INVOLVE advisory group for public involvement in health and care research are upheld as shown in Figure 4.^13^ Those providing insight have the opportunity to see and edit their personal contribution, receive feedback on what has happened after their input was given, and are welcome to be involved in the future iterative development of solutions. This provides two main benefits: Firstly, solutions are likely to be more effective and simplified; Secondly, everybody can see, with full transparency and in practice, that their contribution matters, enabling patients to be active co-researchers in their technology clinical trial.

Iterative development ensures that the new integrated approach addresses the primary research question, whilst also remaining easily achievable for those involved. The iteration-type depends upon the research question being addressed. For example, algorithm development may involve repeat testing with datasets, whereas usability of equipment would need hands-on feedback. In practice, a blend of approaches is usually required with delivery being dependent upon ensuring that all four of the essential elements for health care are addressed:

- Human expertise: Identification of relevant experts, including patients and carers, is driven by developing project requirements, initially explored during DP1 by anyone with relevant insight.
- Science: Research into underlying scientific rationale usually includes scientific literature searches, conference attendance, and discussion within the scientific and clinical research networks. Scientific feasibility work is undertaken in Step 1, with practical application during Step 2.
- Technology: Due diligence activities aim to identify the most appropriate technological elements. This is usually shaped both by what people want, and what technological options are available. The approach must be designed to comply with information governance and General Data Protection Regulation (GDPR) requirements.^17^
- Human behaviour: Established evaluation approaches such as socio-technical systems theory,^18–20^ in connection with psychology-based behaviour-change methodology,^21^ is likely to help scientifically evaluate and structure iterative optimisation of the solution. Additionally, this provides a useful approach for the systematic prediction and mitigation of potential-unintended-consequences that can be measured during the trial.

In addition to functional and data improvements, the ethical considerations of every change in the pathway must be considered. Areas most frequently affected when bringing technology into the clinic are training vs responsibility, and access to solutions. This section therefore focuses on these two areas for discussion.

#### Training vs responsibility

If a solution changes the way people behave and interact there is the tendency to also appear to change responsibilities. For example, a patient has a hospital-based blood test and results inform the doctors treatment decision. If the patient were to self-test at home, and see their results directly, are they now more responsible for their own care? A transparent view of current roles and responsibilities is required before such questions can be addressed.

In this scenario the physician is responsible for the clinical care, interpretation of results, and treatment decisions. The patient is responsible for their own overall wellbeing and actions outside of the clinical environment (they are already responsible for taking their medications and phoning the doctor if they feel unwell). Home testing is likely to require a more involved patient-role in helping their physician, but it does not have to result in the patient taking on responsibility for clinical decisions and actions – this will depend upon how the care pathway is designed.

Although the patient is directly receiving their results, the physician can also have access. In addition, the physician can provide an explicit up-front action-plan at the start of testing so that the patient is always acting on results as per their doctor’s instructions without having to wait or ask every time (e.g. if you miss a test then do X, if the result is over Y then phone the hospital). In this scenario the key ethical debate is less about moving clinical responsibilities and instead focuses on the type of support a participant needs to enable them to undertake new accountability without additional burden.

#### Access to improved solutions

Not everyone carries a mobile phone or likes using technology. Should a non-technical alternative be routinely put into technology trials that, even if it cannot deliver maximum benefit, may hopefully deliver a better standard of care? Fundamentally this will depend upon the research question, an understanding of the population, as well as both the behavioural and technological requirements of the research. However, in general if there is a more familiar (and therefore ‘easy’) option available, human nature in a busy/stressful environment is biased towards people adopting this more familiar approach, even if the potential benefit is reduced (e.g. getting in the car and driving to hospital on Mondays because it's the routine).

The question could then be – is it ethical to provide an alternative that means the solution with maximum potential benefit for patients will never be fully tested in practice? This is a frequent debate, however, until the new care pathway is objectively tested in a technology clinical trial, the hypothesised benefits are unproven. The recommendation is therefore to design the new care pathway for maximum patient benefit and characterise any barriers that may exist in practice and prevent engagement with the new approach during the technology clinical trial. This detailed knowledge can then be used to specifically address the needs of potentially excluded clinical populations and develop methodology that enables broader access when subsequently deployed (e.g. alternatives to allow patients who are technologically illiterate to still enrol in smart-phone driven trials of the future).

### Navigating the regulations

**Figure 5.**
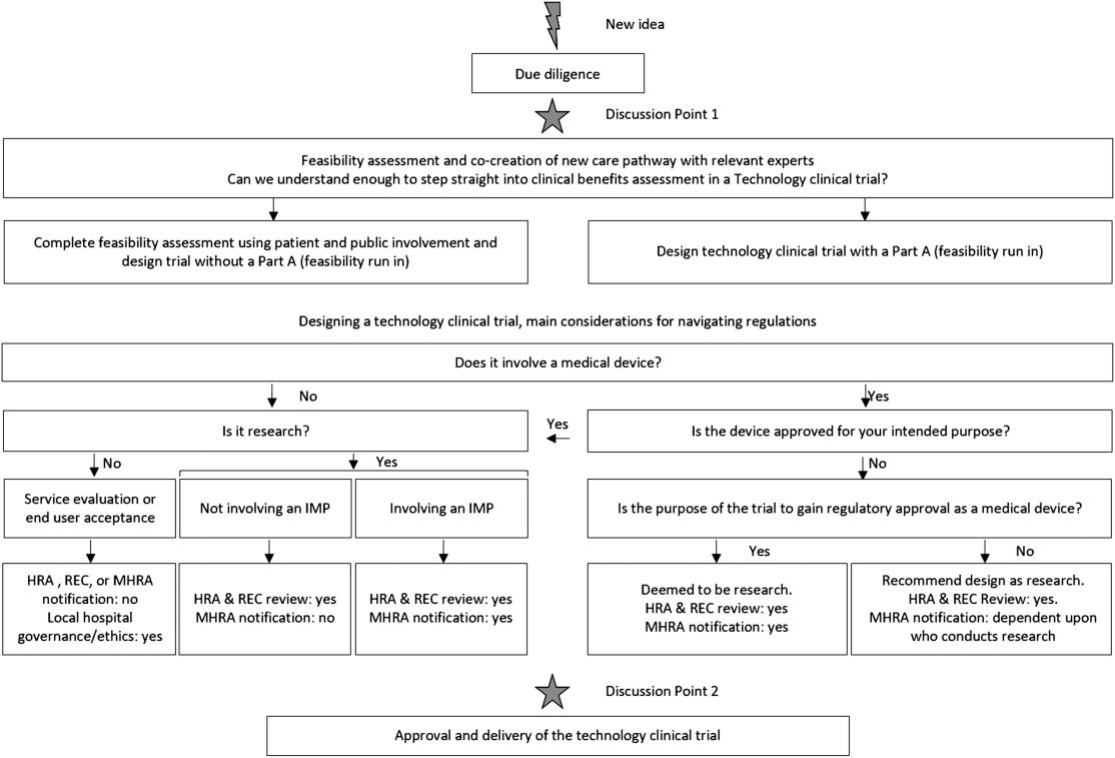
Flow chart to support navigation of regulatory requirements by academic researchers. Flow chart to support research teams when navigating the regulations that may be relevant for a new clinical care pathway. IMP: Investigational medicinal product, HRA: Health Research Authority; MHRA: Medicines and Healthcare Products Regulatory Agency; REC: Research Ethics Committee.

Regulations need to be considered during feasibility and design stages of any approach to ensure that requirements can be seamlessly designed into the solution itself, along with the data security and privacy requirements mentioned above. However, navigation of the regulations for academic researchers is often complicated and confusing. Sometimes a small change to an interface results in researchers being classified as manufacturers of a device, and other times a device is not being tested, or research undertaken, when a solution is rolled out. The methodology described in this article is therefore a simplification of the regulations to support academics when navigating this complex area (Figure 5).

The initial steps outlined in Figure 5 show the due diligence, scientific, iterative, and involvement work described above. Navigation of the regulations is initially an exercise undertaken with each solution iteration during the incubator stage. The first two questions that need to be addressed are whether the piece of work is research, and/or whether it involves a medical device? These appear to be simple questions, but they are not always as easy to navigate as it would initially seem.

#### Is it research?

If participants are current NHS patients, service users, staff or volunteers then the question of whether your solution should be classified as research relates to the Health Research Authority (HRA) requirements.^22^ Fundamentally, this is research if allocation of care or treatment is decided by the protocol or results are generalizable (can be extrapolated to different populations) or transferable (qualitative information that can also be applicable in other settings).

Such ‘research' usually requires assessment of governance and legal compliance (conducted by the HRA) and Research Ethics Committee (REC) review. However, in certain circumstances HRA review is not needed (e.g. research tissue banks or databases, and certain healthy volunteer studies such as Phase I healthy volunteer trials). An even smaller proportion of research does not need REC review (but may still need local review), e.g. usability testing of a medical device by healthy volunteers where sample collection is not required (however, in this scenario these volunteers must not have been selected because of a link with the NHS – an example of which would be carers of patients). The HRA have provided a questionnaire and table to help researchers with these questions.^23,24^ Outside of this scope, other activities are likely to require either service evaluation or audit review within the hospital and, as it has already been decided that this is of local relevance only, results are unlikely to be published in peer-reviewed scientific literature.

The picture is further complicated for academic researchers by the definition of research used by the researcher's affiliated university, which is likely to be broader than that described by HRA. For example, if the research project has randomly selected NHS staff as participants but no tissue sampling is undertaken, this is likely to require HRA, university research and development, and university ethics review (i.e. multiple review rounds), but not NHS REC review. Accidentally designing a trial that requires multiple rounds of governance-review has the potential to hinder research projects. Therefore, navigation of Figure 5 helps researchers to avoid confusion by guiding them to design a solution that clearly fits a single 'box' and expedite the scientific advancement that results may bring.

#### Is it a medical device?

Medical device regulations and guidelines aim to ensure that devices are appropriately assessed for both accuracy and benefit-risk. Therefore, if the proposed solution involves a medical device, these regulations need to be fully understood and their requirements designed into the approach during the initial stages of development. Any part of a system can be classified as a medical device, even software. A comprehensive definition of a medical device can be found in the European Union (EU) Devices Directive, Medical Devices Regulation (MDR) and the In Vitro Devices Regulation (IVDR).^25–27^

In short, a medical device is anything other than drug, immuno- or metabolic- therapy, that is used specifically for diagnostic and/or therapeutic purposes in human beings (including control of conception). Accessories are things which, when used with the device, enable the device’s intended use (e.g. if software runs to produce a calculated output from an electrocardiogram (ECG) that physicians rely upon). Within these definitions there are three main considerations that researchers need to clarify when navigating regulations:

- *Intention for use:* ‘Intention for use' is defined by the manufacturer themselves in their promotional material. Frequently, however, there is doubt about the scope of the advertised intention. Furthermore, the research itself is usually being designed to broaden the use of the device. Clarity is essential and, because there is not one unifying source of approval-scope information, the quickest approach is often to contact the manufacturer and ask directly. If a researcher produces any software to support use of the manufacturer’s product in their research, that software itself could be classified as a medical device or accessory, manufactured by the researcher.
- *Is it an in vitro diagnostic (IVD) device?:* Use of an IVD in a solution needs to be identified as early as possible as IVDs have different regulatory requirements and risk categories to other medical devices. IVDs are anything designed to be used *in vitro* for the purpose of contributing towards a diagnosis. Examples include reagents, controls, containers for human-derived specimens, calibrators. The European Devices Directive contains a full list of definitions and examples and the IVDR contains a full list of definitions that are to be complied with from May 2022.^25,27^
- *Is the software a medical device?:* The Medicines and Health care products Regulatory Agency (MHRA) have produced flow charts to help decide whether software is a medical device.^28,29^ The key question in care-pathway development is usually understanding what is meant by ‘monitoring’ and ‘decision support’. The difference between these essentially rests upon who/what has access to the raw information and makes key decisions. For example, if a clinician uses software that displays all relevant data and maybe adds a colour coding to represent calculated risk, then this is likely to be classified as decision support and subsequently not a medical device. This is because decisions are still made by the expert and that person still has uninhibited access to all the relevant raw data they need – the software is merely supporting the decision-making process. However, if this same clinician were to use a system that displays only results calculated by the software, or an interface that ‘folds’ the information into computer-generated risk-categories, then this is monitoring and likely to be a medical device. In this scenario the computer itself is taking on some of the decision-process either through automated-calculation or through ‘hiding’ the raw data behind pre-specified/defined machine learning algorithms to derive a risk-filtering system for busy clinicians. These considerations can have a major impact on the co-design of software interfaces during feasibility stages of project development. The system should be designed to maximise potential patient-benefit, but the rationale behind whether the resulting approach is ‘monitoring’ or ‘decision support’ needs to be documented and maintained to avoid confusion later. All software that IVDs depend upon for their purpose is automatically categorised as a medical device.

If the research does not contain a medical device, or only contains devices that are being used in accordance to their approved (Conformité Européene [CE]-mark) intended purpose then it is not ‘device research’ and navigation of requirements rests solely on the previous question of whether the activity itself is research.

If a medical device is involved that has not received approval (CE-mark) for the intended use and/or population (called ‘unapproved’ throughout the rest of the article), then the activity should be considered as research involving a medical device. Subsequent requirements for trial design, reporting, and navigation of the regulations depend on the purpose of the research and risk-classification of the device.

#### What is the purpose of the research?

Published guidance for manufacturers is relevant for any trials involving unapproved medical devices when researchers are either working for, on behalf of, a manufacturer.^30^ If the research is being undertaken in the UK, all such research needs to be flagged to the MHRA, plus HRA and REC. For clarity, academics aiming to commercially sell their product (or software produced to work with another product) are considered to be manufacturers. Academics who are conducting research for the purpose of providing the manufacturer with a dataset to support the manufacturer’s submission for medical device approval (CE-mark in Europe), are considered to be working on behalf of the manufacturer. In each of these situations, end-user feedback should be sought during the design phases of the device and tested as part of the trial output. However, separate usability tests or feasibility assessment studies cannot be undertaken until enough data has been gathered to establish the performance and adequate safety of the device by itself when used by relevant populations.^26,27^

Researchers conducting independent off-label work within a single legal entity, and that is not for commercial gain, find themselves in a grey area where notification to the MHRA is not always required. There are two types of work generally undertaken, and both have different considerations: “Feasibility” assessments and full “Technology Clinical Trial” for benefit-risk quantification.

Feasibility assessments are initial ‘try it in practice’ assessments. Technically, feasibility assessments can be conducted without HRA and REC review if they do not meet the NHS/HRA definition of research, and as long as they have local governance review and relevant insurance and indemnity is provided. However, grey-areas such as this often result in confusion and project-delay. Hospitals may struggle to decide which local committee can provide the best governance review. Even if feasibility assessments do not meet the NHS/HRA criteria for research, they are likely to meet university-criteria and therefore full university submission is still likely to be required for feasibility work. In practice, this results in submission to the university for feasibility research, followed by submission to HRA and REC for a separate technology clinical trial. Although possible, the more expedient way to conduct this research is likely to be through designing a single technology clinical trial for submission to HRA and REC with both feasibility and clinical-benefits assessments built into the design.

The final remaining medical device consideration is the level of risk that the device itself poses to humans. This step requires the researcher to evaluate the device based on current knowledge and compare this with standard risk categories for nearly all devices (The Class I to III system).^25,26,31^ As stated previously, IVDs have their own, slightly more complex, classification system involving four categories: 1) General medical devices; 2) Those listed in Annex II List A of the IVD directive; 3) Those listed in Annex II List B of the IVD Directive; 4) Self-tests to be used by a person at home.^27^ There are specific requirements for each category that need to be considered in the design of the planned clinical research, and risk-assessment forms the foundation of mitigation and management activities to protect trial participants.

### Step 2. Technology clinical trial to objectively test the new care pathway

#### Discussion Point 2

At this point in the process a new care pathway has been iteratively co-developed and solutions honed with those involved in the pathway. An estimation of both the size and character of benefits for patients and carers will have been undertaken, and ways to measure these explored. In addition, any specific requirements for research based on navigation of the regulations, ethical considerations, information governance, and privacy have also been clarified and built into the approach. Discussion Point 2 (DP2) is where feasibility is turned into practice.

Research teams have finite resources and therefore only projects with the largest fully quantified and characterised potential for patient benefit proceed past DP2. The aim of a DP2 discussion is to review the research to date and decide the optimal next project steps. Stopping or putting projects on hold is an important aspect of the methodology. If a project is unlikely to provide significant patient-benefit, or is likely to be undeliverable with existing resources, then managing to quantify this and stop the project as quickly as possible frees resources for projects with larger success-potential. This ensures that the research team’s efforts are constantly focused on activities likely to provide most gain for health care, and trials that are thoroughly considered and deemed to be robust in their rationale before commencement.

Of note, even if projects are halted, those who provided input and requested feedback should be kept informed. Many researchers fear patients' reactions when hearing that projects have stopped, but in our experience as long as information is shared transparently and with the full rationale, people have welcomed the news and continued to support the group.

#### Technology clinical trial endpoints

**Figure 6.**
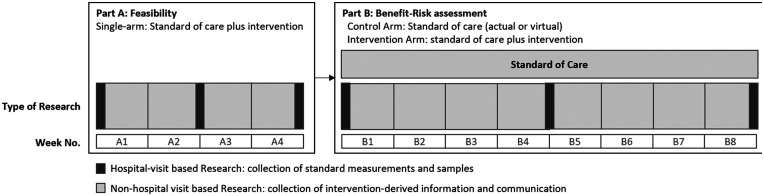
Example technology clinical trial design. Example trial design with Part A and Part B. Duration, monitoring requirements, and outcome measures for the trial will be defined during feasibility work for the research question.

Establishing and testing a new clinical trial care pathway is complex and requires a wider range of endpoints than would normally be collected within a CTIMP study. Therefore, the Medical Research Council (MRC) guidance for developing and evaluating complex interventions has been used to design the experimental approaches described within this document.^32^ One of these is to include a ‘Part A: Feasibility' that is a short, in-practice feasibility assessment where participants use the approach and provide feedback. The importance of this is that practical application often uncovers aspects of a process that previous iterative feasibility does not. If these exist, it is important to uncover them with a small number of participants during Part A so that improvements and risk mitigation can be put in place before the larger Part B: Benefit-Risk Assessment. Part B aims to measure the clinical benefits of the optimised approach when compared to standard of care (via a control study-arm, or a virtual control group from relevant record data; Figure 6).

The aim is to also change the way in which medical teams and participants interact, contribute, and use data. The National Institute for Health and Care Excellence (NICE) Behavioural change evaluation guide therefore provides an excellent research structure that can be delivered within the MRC framework.^33^ This structure ensures that there is a systematic, measured, empirical approach consistently applied across all endpoints to cover the effectiveness, acceptability, feasibility, equity, and safety of the whole intervention.

#### Trial governance and decisions

Each study should have a steering committee providing oversight that consists of relevant experts and the chief investigator of the trial. The Part A-B juncture should trigger an interim review and/or analysis to assess all feasibility data from Part A plus all feedback information received. The steering committee assess whether the primary research question is being addressed optimally in practice. The exact nature of this assessment will be driven by the research question itself. However, as a general rule factors reviewed to help inform this decision will include recruitment rate (actual vs expected rate); adherence to required readings (more or less than 75 percent if readings are taken); ethical assessment as per primary objective after in-practice learning (generalizability, availability of the intervention, and assessment of unintended consequences).

A red/amber/green (stop/amend/continue) traffic-light approach could be applied to the criteria used to determine whether to progress with Part B: Benefit-Risk Assessment.^34^ If progression criteria are not met and cannot be mitigated (usually using Part A: Feasibility feedback as a guide), then the study should not progress beyond this point and the clinical study report will be authored to reflect this. However, if criteria are met or, in the opinion of the steering committee can be met, the study will progress onto Part B for clinical benefits assessment classification as shown in Figure 6. Any changes to the study documentation must be re-reviewed by the relevant authorities, ethical bodies, and governance committees as per regulatory requirements.

### Step 3. Sharing and use of the data to further research and clinical care

Technology clinical trials frequently aim to improve the effectiveness of people’s health care relationships and ownership of their wellbeing. Therefore, careful consideration should be undertaken when writing up the study and circulating both individual and trial results. In addition to the clinical study report (CSR) produced for ethics committees and authorities, each participant should have the opportunity of reviewing their own data, as well as the consolidated results from the trial. This normally means writing up a lay-person version of the clinical study report and submitting back to ethics committee at the same time as the CSR with a request that this can be sent to all participants who are still contactable.

In addition, researchers should consider availability of data in the scientific literature. If the technology clinical trial aims to empower participants, and clinical scientists are asking them to take on a more active role in the research, participants should be acknowledged in any subsequent publications and open-access publishing should be considered so that participants and their families do not face a pay-wall when trying to access the information that they contributed towards.

The ultimate aim of this research is to characterise the benefits, risks, and any unforeseen effects of a new, integrated care pathway. These data should therefore also be shared back with the service evaluation team at the hospital for consideration for improvements in normal standard of care approaches and circulated among the clinical trial communities for advancing clinical trial practice. Data gathered during exploratory objectives frequently highlight areas of improvement in the process or new research opportunities with the potential to further enhance patient benefit. These new hypotheses are explored during new DP1 meetings with relevant experts from the research team and community to assess the potential gains in patient benefit this new research may bring.

## Conclusions

Although technology has the potential to revolutionise clinical trials and improve patient benefits, the potential for transformative change has, until now, not been fully realised. This is in part due to a traditional approach focusing on the benefit-risk of specific novel drugs and/or devices in clinical trials, without testing the broader benefits and risks that arise when the whole care-pathway is optimised. It has also been in part due to the research groups not having the clear methodology to undertake their co-creation of care pathways within the hospital environment, navigate the complex regulatory landscape, and build capability to deliver trials that objectively test complex interventions spanning technology, process, data and behavioural science.

The method described in this article has been successfully used in practice, and provides a mechanism to navigate the barriers described, offering an approach for objective integration of technology into the cross-disciplinary movement of evidence-based medicine. The technology clinical trial has the potential to break the deadlock that researchers are currently facing and accelerate the integration of new science and technology into practical care pathways delivering optimised benefits for patients.

## Abbreviations

CE-Mark: Conformité Européene mark

CSR: Clinical study report

CTIMP: Clinical trials of investigational medicinal product

DP1: Discussion point 1

DP2: Discussion point 2

ECG: Electrocardiogram

digital ECMT: digital Experimental Cancer Medicine Team

EU: European Union

GDPR: General Data Protection Regulation

HRA: Health Research Authority

IMP: Investigational medicinal product

IVD: In vitro diagnostic

IVDR: In Vitro Devices Regulation

MDR: Medical Devices Regulation

MHRA: Medicines and Healthcare Products Regulatory Agency

MRC: Medical Research Council

NHS: National Health Service

NICE: National Institute for Health and Care Excellence

NIHR: National Institute for Health Research

REC: Research Ethics Committee
